# The Role of Correction in the Conservative Treatment of Adolescent Idiopathic Scoliosis

**DOI:** 10.2174/1874325001711011548

**Published:** 2017-12-29

**Authors:** Shu-Yan Ng, Xiao-feng Nan, Sang-Gil Lee, Nico Tournavitis

**Affiliations:** Wanchai Chiropractic Clinic, 11/fl China Hong Kong Tower, 8 Hennessy Road, Wanchai, Hong Kong

**Keywords:** Adolescent idiopathic scoliosis, Correction, Scoliosis specific exercises, Pattern-specific scoliosis rehabilitation, Physical therapy, In-brace correction, Brace

## Abstract

**Introduction:**

Physiotherapeutic Scoliosis-Specific Exercises (PSSE) and bracing have been found to be effective in the stabilization of curves in patients with Adolescent Idiopathic Scoliosis (AIS). Yet, the difference among the many PSSEs and braces has not been studied. The present review attempts to investigate the role of curve correction in the outcome of treatment for PSSEs and braces.

**Material and Methods:**

A PubMed manual search has been conducted for studies on the role of correction in the effectiveness of PSSE and bracing. For the PSSEs, the key words used were “adolescent idiopathic scoliosis, correction, physiotherapy, physical therapy, exercise, and rehabilitation.” For bracing, the key words used were “adolescent idiopathic scoliosis, correction and brace”. Only papers that were published from 2001-2017 were included and reviewed, as there were very few relevant papers dating earlier than 2001.

**Results:**

The search found no studies on the role of correction on the effectiveness of different PSSEs. The effectiveness of different PSSEs might or might not be related to the magnitude of curve correction during the exercises. However, many studies showed a relationship between the magnitude of in-brace correction and the outcome of the brace treatment.

**Discussion:**

The role of correction on the effectiveness of PSSE has not been studied. In-brace correction, however, has been found to be associated with the outcome of brace treatment. An in-brace correction of < 10% was associated with an increased rate of failure of brace treatment, whereas an in-brace correction of >40-50% was associated with an increased rate of brace treatment success (*i.e*. stabilization or improvement of curves). Thus, in the treatment of AIS, patients should be advised to use highly corrective braces, in conjunction with PSSE since exercises have been found to help stabilize the curves during weaning of the brace. Presently, no specific PSSE can be recommended.

**Conclusion:**

Braces of high in-brace correction should be used in conjunction with PSSEs in the treatment of AIS. No specific PSSE can be recommended as comparison studies of the effectiveness of different PSSEs are not found at the time of this study.

## 1 INTRODUCTION

Adolescent idiopathic scoliosis (AIS) refers to the lateral curvature of the spine in excess of 10^o^ in patients aged 10-18 with no identifiable causes. For curves of Cobb angle between 20-40 degrees, conservative treatment is indicated [1, 2]. According to the SOSORT guidelines, this includes the physiotherapeutic scoliosis-specific exercises (PSSE) and spinal bracing [1, 2].

Despite the recommendation, the use of PSSE and spinal bracing in the management of AIS has been controversial [3, 4]. Recently, their effectiveness has been proven by randomized controlled trials (RCT) [5-7]. At present, PSSE and spinal bracing have level I evidence in support of their effectiveness. Monticone *et al.* (2014) reported that active self-correction could improve the Angle of Trunk Rotation (ATR) and Cobb angle of AIS patients [5]. Kuru *et al.* (2015) and Schreiber *et al.* (2016) have reported that the Schroth Best Practice approach (out-patient Schroth approach) and the traditional Schroth approach, respectively, could stabilize and improve the curves of AIS patients [6, 7]. Also, Weinstein *et al.* (2013) reported that TLSO braces were able to reduce progression of curves to below the threshold of surgery in 72% of the AIS patients in a randomized and preference controlled trial [8].

These studies support both PSSEs and spinal bracing in the treatment of AIS. Nevertheless, whether the outcome can be generalized to all PSSEs and braces remain to be studied as there are many different types of PSSEs and braces which have different principles of correction.

To improve the outcome of conservative treatment of AIS, we reviewed the literature to determine if the degree of correction during treatment plays a role in the outcome of PSSEs and brace treatment of AIS.

## 2 MATERIAL AND METHODS

We conducted a PubMed search using the keywords “AIS, idiopathic scoliosis, correction, physiotherapy, physical therapy, exercise and rehabilitation“ for the PSSE and “AIS, correction and braces“ for the braces. Only papers that were published in the last 15 years were included. We excluded articles on congenital scoliosis, juvenile idiopathic scoliosis and syndromic scoliosis. Also, articles on pathology, etiology, and biomechanical principles were excluded. We did not include articles on night bracing, as night braces involve hypercorrection and direct comparison of in-brace correction with other full-time braces is not appropriate.

For PSSEs, papers that complied with SOSORT guidelines were included. For bracing, we only included studies that complied with the Scoliosis Research Society (SRS) bracing requirements, and those which included patients who were reportedly compliant with brace treatment.

## 3 RESULTS

For the PSSEs, the PubMed search generated a total of 90 papers. Seventy-six papers were excluded, as they were either not in English or the contents were not relevant to the present discussion. This left only 14 papers, which included the three randomized controlled trials [5-7].

### 3.1 Role of Correction in PSSE/PSSR

**Fig. (1):**
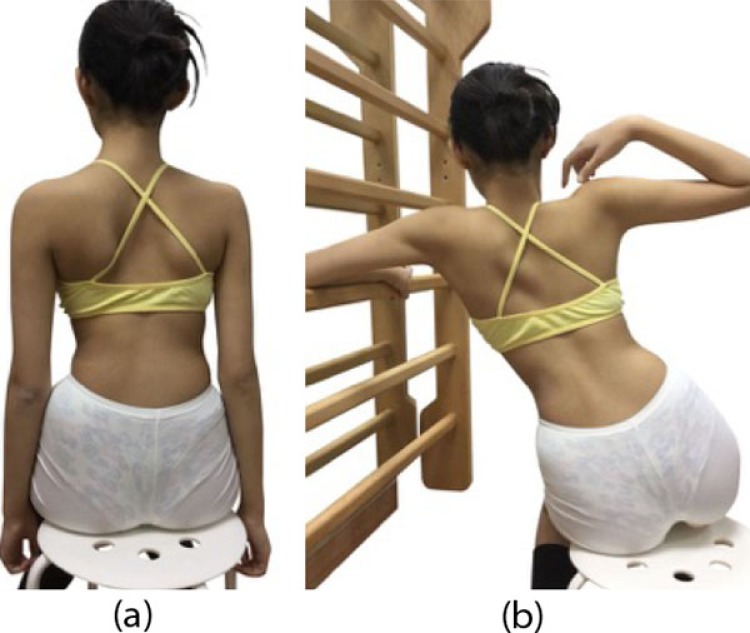
The Handle Exercise. (a) The patient presented with a right thoracic scoliosis. (b) Side shifting to left overcorrected the thoracic scoliosis.

A review of the papers showed that at present, there are eight different schools of PSSEs. They are namely the Barcelona Scoliosis Physical Therapy School (BSPTS), Dobomed, FITS (Functional Individual Treatment to Scoliosis), the Lyon approach, Schroth Scoliosis Intensive Rehabilitation, Schroth Best Practice (out-patient approach), SEAS (Scientific Exercises Approach to Scoliosis) and the side-shift [9, 10]. They have different principles of treatment. The BSPTS and the Schroth Best Practice are derivatives of the original Schroth method. All the three Schroth approaches use corrective or over-corrective movements for exercise treatments (Fig. **1**). The corrective exercises used are pattern specific and can be described as pattern specific scoliosis rehabilitation (PSSR). During exercises, the patient is instructed to shift their trunk past the neutral position into the opposite direction [11]. The side-shift approach uses similar corrective or over-corrective movements. All other PSSEs, including Dobomed, FITS, Lyon approach and SEAS move the spine to the neutral position [9]. Thus, there are two different approaches to correction, one involving over-correction and another involving correction to a neutral position. Whether these approaches bring about different outcomes has not been studied as yet. We were unable to identify any papers studying the difference in the outcome between these two approaches. The role of correction in the effectiveness of PSSEs is not clear.

### 3.2 Role of Correction in Bracing Outcome

The PubMed search generated 261 papers published from 2001 to 2017. Of these, 26 were not in English. Papers that were related to early onset scoliosis, syndromic scoliosis, surgery, biomechanics and treatment of large curves were excluded. This left 77 papers for review. Only studies that complied with the SRS guidelines were reviewed [12]. The SRS guidelines stipulated that bracing should only be prescribed for patients aged 10 or above, with Risser 0-2 and primary curve angles 25^o^-40^o^. When the patients are female, they should be either premenarchal or less than 1 year postmenarchal [12].

The review showed that in-brace correction was related to the success of brace treatment [13-16]. Of the relevant papers, we identified five papers that included data on in-brace correction and treatment outcome 1-2 years after brace weaning (Table **1**). Though there were only a small number of papers, the results suggest that a high in-brace correction was associated with a high rate of treatment success (stabilization and/or improvement of curves).

## 4 DISCUSSION

From our literature review, it was apparent that there was no research that investigated the relationship between correction and treatment outcome for the various types of PSSEs. This might be due to the difficulty of measuring correction during the exercises. X-rays have not been used to quantify the magnitude of correction during exercises. It would be unethical to do so because of the radiation exposure to the patient. Also, different specialized scoliosis centers use different approaches, making outcome comparison difficult.

Due to the fact that the three Randomized Controlled Trials (RCTs) used active self-correction and the Schroth out-patient approach as interventions for AIS patients [5-7], we could only regard PSSEs with active-self correction and Schroth out-patient approaches as effective intervention in the treatment of AIS. The methods used in the three RCTs follow a certain routine based on a classification of curve patterns and well-described patterns of correction [5-7]. All the other exercise programs such as FITS, Dobomed and SEAS, are not presently supported by high-quality evidence.

In the RCTs investigating high-correction exercises [5-7], the outcome parameter used was progression rate comparing two different types of treatments [5, 7]. However, only the Kuru *et al.* (2015) paper presented an untreated control group [6].

PSSE is a general term used for all exercise-based scoliosis treatment approaches. Therefore, it does not seem appropriate to claim that high-quality evidence exists for all the different PSSE approaches currently used [9].

The amount of correction (as a percentage of the initial Cobb angle) as a result of specific exercises has not been described in an end-result study. However, in a short-term prospective cohort study using the Schroth Best Practice® PSSR approach, a subset of patients with an initial Cobb angle over 30° were able to achieve an average of 9.3 degree improvement over a 3-month period of time [13].

The correlation between in-brace correction of Cobb angle and the final bracing outcome was more clearly established in the literature [14-16]. In an attempt to determine a concrete cut-off point to predict success or failure of bracing, Xu *et al.* (2015) followed 488 AIS patients until two years after weaning of the Boston brace treatment. They found that an in-brace correction of less than 10% was associated with a higher rate of brace failure (>5° increase of Cobb angle) compared to an in-brace correction of more than 10% [17]. In-brace correction of at least 20% has been found to prevent progression of curvatures [16]. Correction of 30% or more is required to achieve final improvement when skeletally mature [15, 18]. In the latter studies, improvement was defined as a reduction of >5^o^ Cobb angle. Landauer *et al.* (2003) found that an in-brace correction greater than 40% was associated with an improvement of 7° upon cessation of treatment [16]. Similarly, Olafsson *et al.* (1995) found that an in-brace correction of more than 50% was associated with a mean improvement of 7.2 degrees upon termination of brace treatment [14].

Yamane *et al.* (2016) have shown that the correction of vertebral rotation is also an important factor in brace treatment outcome [19]. In patients with Lenke type 1 curve, insufficient correction of vertebral rotation was associated with a higher rate of brace failure (failure being defined as any of the following: >5° increase of Cobb angle, >5° increase in rotation, or patient needing surgery), whereas greater correction of vertebral rotation was associated with a higher rate of brace treatment success [19].

These studies suggest the need for a large in-brace correction to achieve final improvement once the patient is skeletally mature. These findings were supported by other studies that complied with the SRS bracing guidelines [12]. The studies shown in Table **1** included data on in-brace correction of Cobb angle as well as treatment results 1-2 years after brace weaning. A good in-brace correction was associated with an increase in the success rate, which was defined as the sum of improvement and stabilization rates. Improvement of curves refers to a reduction of curves of >5^o^ and stabilization of curves refers to a Cobb angle of ± 5^o^ as compared to the baseline. Patients wearing the Rosenberg brace had an average in-brace correction of 30% [20]. Only 43.7% of the patients achieved stabilization of their scoliosis while 56.3% of the patients had worsening of the curves by >5^o^. In the study, patients whose curves did not progress by more than 5^o^ and those who did not have surgery had a mean in-brace correction of 37% and 33%, respectively.

**Fig. (2):**
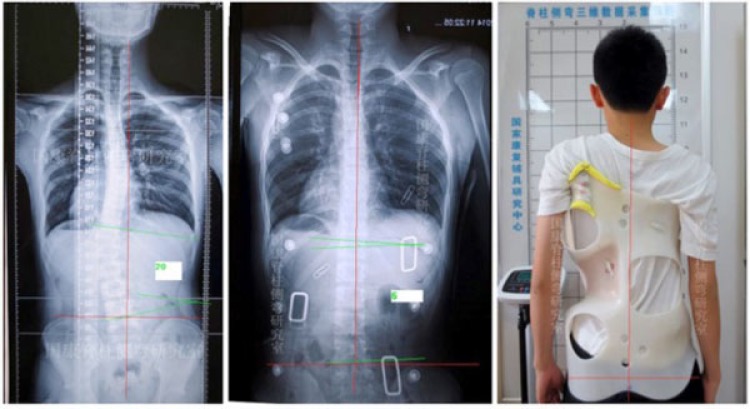
Cast based Chêneau brace with nearly full correction of a double major curve pattern. This ‚old style‘ Chêneau brace has been produced in China (with kind permission by Xiaofeng Nan, Xi‘an).

Patients who experienced curve progression of > 5^o^ and those who required surgical intervention had an in-brace correction of 22% and 21%, respectively [20]. The study by Yrjönen *et al.* (2007) showed better results using the Boston brace [21]. For the 33 male AIS patients (mean age of 14.9 years), the average in-brace correction was 40% and 81.8% of the patients had their curves stabilize. The stabilization or success rate was slightly reduced to 72.7% for the younger female patients [21]. The Lyon brace had an average in-brace correction of 63% and a success rate of 85% [22]; however, it is noteworthy that the Lyon brace is generally fitted on the patient after he or she is put in plaster cast correction for 1-4 months. The patients wearing the Chêneau brace (Fig. **2**) had an average in-brace correction of 72% and a success rate of 100% [23]. In the light of these results, it appears that a better in-brace correction correlates with a better success rate. However, the results of the studies by de Mauroy *et al.* [22] and de Giorgi *et al.* [23] are skewed by the fact that the patients were also following an exercise regimen., the combined effect of bracing and PSSE versus bracing alone warrants further study.

**Fig. (3):**
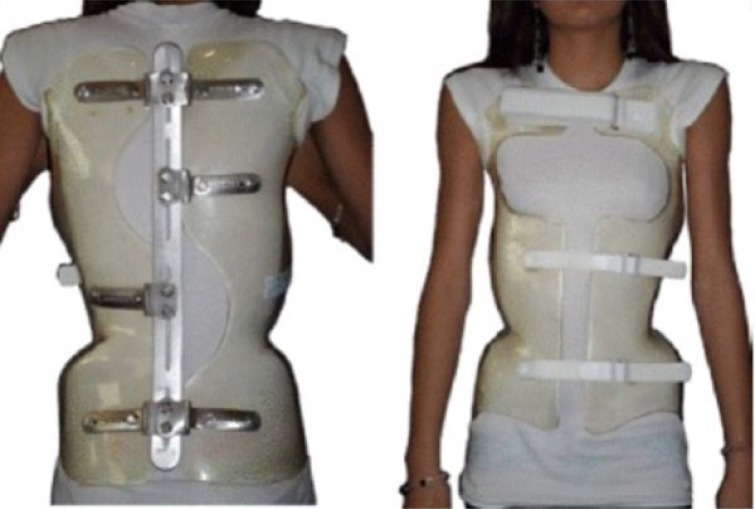
The ART Brace (published in: https://www.ncbi.nlm.nih.gov/pubmed/27525315).

**Fig. (4):**
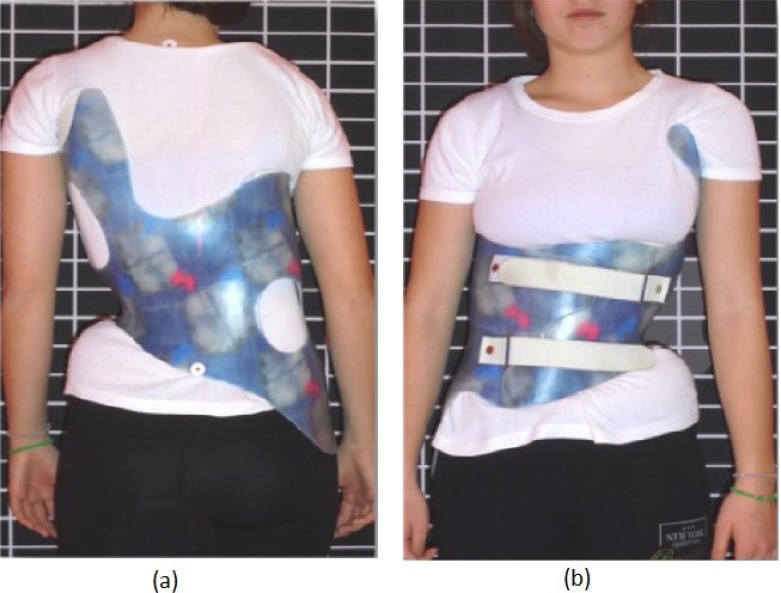
The Gensingen Brace (GBW) for the same curve pattern (Double Major) as the ART Brace on Fig. (**2**). This brace is considerably smaller than the braces on Figs. (**1** and **2**). (With kind permission by Nico Tournavitis, SBPRS, Thessaloniki, Athens, Nicosia).

**Fig. (5):**
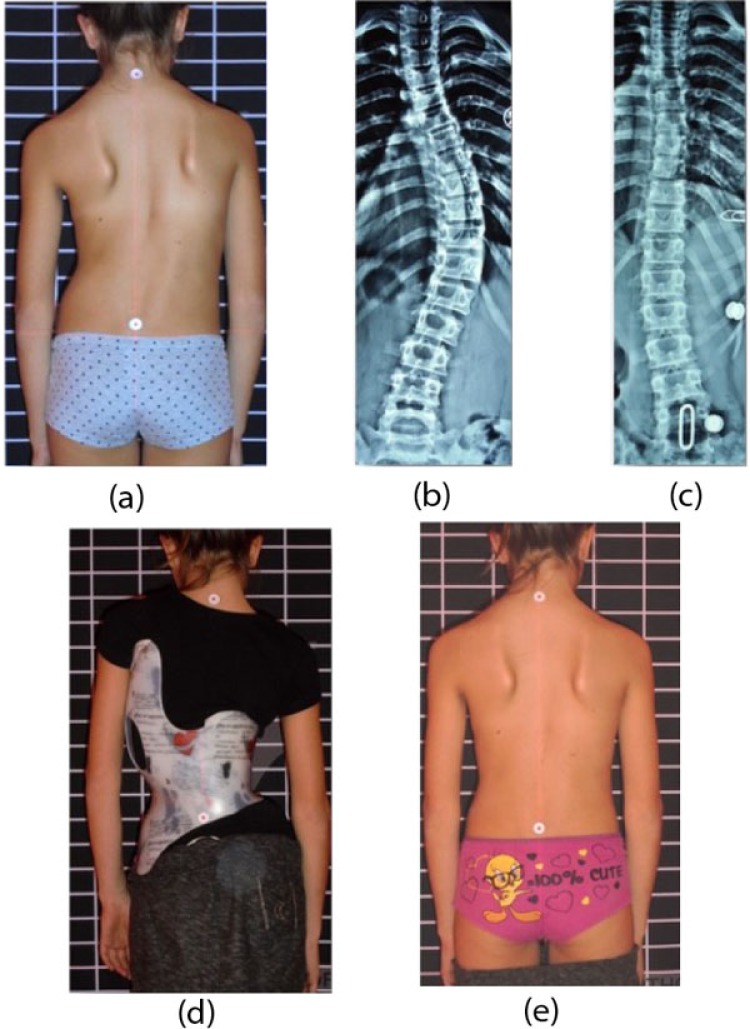
In single curve patterns the deformity mirroring effect is clearly visible and a recompensation will be the result when the brace is worn. This Gensingen brace was made in Greece (With kind permission by Nico Tournavitis, SBPRS, Thessaloniki, Athens, Nicosia).

Other studies have reported even higher percentages of in-brace correction. Presently, the highest average in-brace correction reported for a full-time brace is for the ART brace (Asymmetric, Rigid, Torsional) (Fig. **3**), which is a new version of the Lyon brace [24]. De Mauroy *et al.* (2016) reported the outcome of using the ART brace on 544 AIS patients, 85% of whom were girls. The average in-brace correction was 79.4% of initial Cobb angle. Post-weaning, the patients had a 50% reduction in Cobb angle out-of-brace [24]. The Chêneau-Gensingen brace (Figs. **4**, **5**) and the Rigo System Chêneau (RSC) brace ranked second and third at 66% [25] and 53.8% average in-brace correction respectively [26]. Though these studies didn’t include results two years post-weaning, it is likely that compliant patients will have a good outcome upon termination of bracing. De Mauroy *et al.* (2016) reported a mean weaning correction rate of over 50% [22]. In addition, the success rate of the Chêneau-Gensingen brace has been reported to be in excess of 90% in other studies [27, 28], with no patients going on to require surgery [27]. Weiss (2014) reported a case of an adolescent girl with idiopathic thoracic scoliosis of 38° treated with the Chêneau-Light brace (predecessor of the Cheneau-Gensingen brace) at the age of 11 [29]. At the age of 21, six years after weaning off of the brace, the patient’s curves measured only 19° (50% reduction), suggesting that lasting curvature reduction is possible with bracing [29].

Based on the studies included in this review, it is evident that in-brace correction plays a major role in the final outcome of brace treatment. To improve the outcome of treatment, a brace with the best possible in-brace correction should be used. Preferably, patients should perform PSSE/PSSR in conjunction with brace treatment, as they have been shown to improve the outcome of treatment [30] and to avoid loss of correction after brace weaning [31].

According to Landauer *et al.* (2003), in-brace correction and compliance determine the outcome of brace treatment [16]. The matter of brace-wearing time as the predictor of success was also reflected in a meta-analysis [32]. Therefore, in an effort to improve compliance and brace treatment success rates, efforts have to be taken to make braces highly corrective, smaller, lighter, and more comfortable for patients to wear on a full-time basis. Ward and colleagues (2016) supported the idea of improving and expanding conservative approaches, even to patients with curvatures exceeding 40° during growth [33].

## CONCLUSION

Not all PSSE/PSSR programs are supported by high quality evidence, but active self-correction and Schroth exercises are supported by Level 1 evidence. Whether all PSSEs are equally effective in the treatment of AIS has yet to be determined, as we were unable to identify any studies that investigated or compared the outcome of different PSSEs. We were thus unable to draw any conclusion about the role of correction in the outcome of PSSE treatment.

Conversely, braces with high in-brace correction averages have been found to be associated with good outcomes. One study found that an in-brace correction of >40% improved the curves by 7^o^ at the termination of treatment. Thus, in clinical practice, only braces with high in-brace correction should be prescribed, with the objective of improving or stabilizing the curve.

## Figures and Tables

**Table 1:** The in-brace correction and the outcome of bracing in different studies following the SRS (2005) guidelines. RSC refers to Rigo System Cheneau brace. Cheneau + ex refers to Cheneau brace and PSSE. T1 (B) refers to baseline Cobb angle before bracing. T2 refers to Cobb angle after bracing. % corr refers to the percentage of in-brace correction. Imp% refers to percentage of improvement of curves by more than 6^o^; stab % refers to curves change within ± 5^o^ of baseline measurement; worse % refers to percentage of curves increase by 6^o^ or more. Surg % refers to percentage of patients that required surgery for treatment. NR refers to “not reported”.

Brace	Authors	Age	No/Sex	T1 (B)	T2	% Corr	Imp %	Stab %	Worse %	Surg %
Rosenberg	Spoonamore *et al.* 2004	10 to 16	59F12M	30.9	21.5	30	-	43.7	56.3	31
Boston	Yrjönen *et al.* 2007	14.9	33M	31.5	18.8	40	-	81.8	18.2	6
Boston	Yrjönen *et al.* 2007	13	33F	31.9	16.8	47	-	72.7	27.3	NR
RSC	Maruyama 2015	11.9	27F, 6M	30.8	14.2	54	24.2	51.6	24.2	12
Lyon	de Mauroy 2011	13.8	1338	29.5	10.8	63	67.2	27.8	5	NR
Cheneau + ex	De Giorgi 2013	11.3	48	27	7.6	72	100		0	0
